# Real-Time UAV Autonomous Localization Based on Smartphone Sensors

**DOI:** 10.3390/s18124161

**Published:** 2018-11-27

**Authors:** Boxin Zhao, Xiaolong Chen, Xiaolin Zhao, Jun Jiang, Jiahua Wei

**Affiliations:** 1 Equipment management and UAV Engineering College, Air force Engineering University, Xi’an 710038, China; zhaoxiaolin00@mails.tsinghua.edu.cn (X.Z.); jiang_mail@163.com (J.J.); weijiahua@126.com (J.W.); 2 Science and Technology on Aircraft Control Laboratory, FACRI, Xi’an 710038, China; nxcxl@aliyun.com

**Keywords:** smartphone sensors, UAV autonomous localization, multi-sensor fusion

## Abstract

Localization in GPS-denied environments has become a bottleneck problem for small unmanned aerial vehicles (UAVs). Smartphones equipped with multi-sensors and multi-core processors provide a choice advantage for small UAVs for their high integration and light weight. However, the built-in phone sensor has low accuracy and the phone storage and computing resources are limited, which make the traditional localization methods unable to be readily converted to smartphone-based ones. The paper aims at exploring the feasibility of the phone sensors, and presenting a real-time, less memory autonomous localization method based on the phone sensors, so that the combination of “small UAV+smartphone” can operate in GPS-denied areas regardless of the overload problem. Indoor and outdoor flight experiments are carried out, respectively, based on an off-the-shelf smartphone and a XAircraft 650 quad-rotor platform. The results show that the precision performance of the phone sensors and real-time accurate localization in indoor environment is possible.

## 1. Introduction

### 1.1. Motiviation

In recent years, small unmanned aerial vehicles (UAVs) have shown great application potential in the field of rescue and relief work [1,2], environment exploration [3,4], and so on, for their light weight and agile movement. At present, small UAVs are mainly used in wide open outdoor environments, depending on their built-in GPS modules. However, with the expansion of its application field, small UAVs will be faced with the challenging environment where the GPS signal is unstable or even missing, such as in the interior, tunnels, woods, or mountainous areas in the future, so small UAVs are required to sense the ambient environment autonomously by using their built-in sensors [5,6]. Currently, vision sensors, laser scanners, accelerometers, and gyroscopes are common position sensors that can be used to locate. However, considering the limited load capacity and the cost of the small UAV, unlike unmanned ground vehicles, it cannot be equipped with equipment of high precision and large volume. Hence, low-powered, light, and cheap micro-electro-mechanical system (MEMS) sensors are preferred alternatives.

A mobile phone, the platform that combines multi-sensors and multi-core processors, has high integration, small volume, and light weight. With the development of the mobile phone, the type of built-in sensors and the power of processors are continuously advanced and enhanced. If the smartphone could be used as the airborne sensor for the small UAV, and the localization algorithm is implemented based on the mobile device, then the combination of “small UAV+smartphone” can operate in the interior or small areas without a GPS signal regardless of the overload problem. Therefore, the high integration and improving performance of the mobile phone make it advantageous for the airborne equipment of the small UAV. However, it faces great challenges mainly from the following two points:

1. The built-in MEMS phone sensor is low-priced, and its accuracy is relatively low. The measurement data contains a large amount of noise, which can seriously affect the sensor for accurate calculation, such as UAV localization. At present, the common standard and testing methods have not been formulated in the research field on these MEMS sensors [7], so the first question is supposed to determine which sensors in mobile phones can be used for UAV localization.
2. The limited storage and computing resources of the mobile phone pose another challenge. After years of exploration and practice in the academic field, visual information has been outstanding in solving the problem with the UAV location, typically such as visual odometry (VO) [8,9,10] and visual simultaneous localization and mapping (VSLAM) [5,11,12], which have made amazing achievements. However, the operation of these algorithms requires sufficient storage space and computing resources to store environment maps, loop closure detection, and local/global optimization, which challenge the performance of the mobile phone.

### 1.2. Contributions

In view of the above challenges, this paper expanded our previous work [13] and the following two contributions were made:

1. Accuracy calibration experiments were designed firstly, and the feasibility and precision of the mobile phone sensors (accelerometer, gyroscope, pressure sensor, and orientation sensor) were evaluated.
2. An efficient and real-time autonomous localization algorithm was designed based on the off-the-shelf smartphone sensors. Indoor and outdoor flying experiments were carried out to verify the accuracy of the proposed approach.

## 2. Related Work

Modern smartphones equipped with multiple MEMS sensors, such as accelerometer, gyroscope, camera, pressure sensor and GPS receiver, which measure motion, orientation and various environment conditions, create opportunities for using these mobile devices for location-based applications. In 2014, a project named “Tango” [14,15] was proposed by Google to give mobile devices a human-scale understanding of space and motion, without using GPS or other external signals. Chen et al. [16] proposed a sensor fusion localization framework for combining WiFi, pedestrian dead reckoning, and landmarks based on a smartphone. In addition, Bo et al. [17] presented a system named SmartLoc to estimate the location and the travelling distance of a vehicle in urban environments by using smartphones. Yang et al. [18] proposes a cost-effective method for 3D thermal model reconstruction based on image-based modeling using phone sensors. These algorithms were mainly developed for phone users [14,15,18] or ground vehicles [17].

On the other hand, an increasing number of research works have studied to combine the small UAV and a smartphone. The first combination of UAV and smartphone was in 2010 [19]. A Nokia N95 mobile phone was used as an onboard image processor for quadcopter self-localization. An image database of the environments was established and the quadcopter was able to locate itself by comparing a current image taken by the phone with earlier images in the database. In 2012 and 2013, Yun et al. [20,21] assessed the possibility of smartphone as a payload for photogrammetric UAV system. A smartphone-based photogrammetric UAV system application was developed, through which real-time image, location, and attitude data was obtained using smartphone under both static and dynamic conditions. Then between 2014 and 2018, the phone-based UAV system began to emerge [22,23,24,25]. In 2014, Leichtfried et al. [22] presented the SmartCopter system, which is a low-weight and low-cost UAV that can navigate in GPS-denied environments using an off-the-shelf smartphone as its core on-board processing unit. Then, at the 2015 IROS, Vijay Kumar’s group published a paper [23] which presented a fully autonomous smartphone-based quad-rotor. All the computation, sensing, and control were conducted on an off-the-shelf smartphone, with all the software functionality in a smartphone app. In recent years, a growing number of researchers turned to study the micro aerial vehicle (MAV) control problem based on phone sensors. Astudillo et al. [24] designed an Android-based cascade PID controller structures to control the altitude and attitude of a quadcopter based on phone sensors. Pedro et al. [25] analyzed the accuracy of several localization algorithms in a remote location using smartphones. Wu et al. proposed an automatic and continuous radio map self-updating service for wireless indoor localization by using smartphones [26]. Zhang et al. presented a migratory birds-inspired navigation system integrating phone sensors and the landmarks GPS information [27].

Among the above studies, the core ideas of the papers [22] and [23] are quite similar with ours but the research focuses are different. The two papers mainly focus on the on-board hardware implementation and flight controlling, while the mapping and localization algorithm was chosen from the existing approaches, which integrates the VO/VSLAM algorithm and IMU sensor measurements based on a filter framework. Current VO and VSLAM algorithms [5,8,12] need to store the environment map, aiming at loop closure detection and camera poses optimization, which are now mainly used in ground vehicles. However, if the implementation is based on mobile phones under the condition of limited storage space and computing resources, the related optimization strategy will greatly reduce the real-time of the algorithm and fail to quickly provide location information of the platform. Our paper mainly focuses on exploring the feasibility and accuracy performance of the phone sensors, figuring out which sensor in the phone can be used in an accurate computation, and designing a localization algorithm without constructing the environment map. The localization algorithm requires less storage which is more suitable for the UAV-phone combination and the localization accuracy was compared with the algorithm proposed in [28].

The rest of the paper is organized as follows: Section 3 assesses the availability of phone sensors and designs an autonomous localization approach based on the assessment results. Section 4 presents the experimental evaluation of our proposed approach. Section 5 discusses the future work and concludes the paper.

## 3. Materials and Methods

### 3.1. Mobile Phone Sensor Performance Evaluation

The mobile phone sensor coordinate frame takes the center of the mobile phone as the origin, and the *X* axis and the *Y* axis are, respectively, parallel to the long and short edge of the phone, and the *Z* axis is perpendicular down to the mobile phone screen. The phone’s built-in accelerometer, gyroscope, and orientation sensor readings are measured based on the phone sensor coordinate frame.

#### 3.1.1. Gyroscope

According to [29], the measurement value of the gyroscope sensor is modeled as:

(1) $$\omega_{r}=K_{\omega }\omega_{m}-b_{\omega }-w_{\omega }$$

where $\omega_{r}$ is the real angular velocity of the sensor; $\omega_{m}$ is the measurement value; $K_{\omega }$ is the installation error matrix of the gyroscope; $b_{\omega }$ is the bias; and $w_{\omega }$ is the measurement error of the sensor.

**Figure 1:**
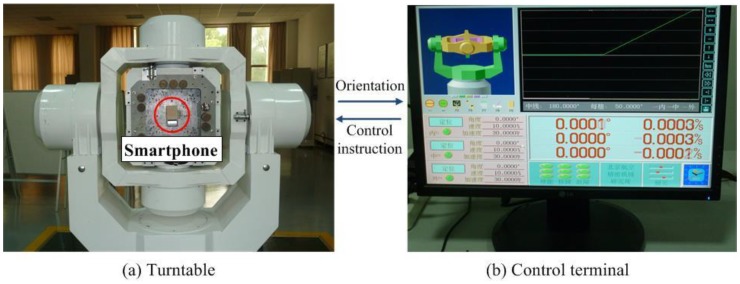
The turntable for phone sensor accuracy estimation.

According to Equation (1), it can be observed that if a gyroscope is available, its bias should be constant (can be modeled) and repeatable. In order to verify the bias performances, the mobile phone was installed in the center of a three-axis turntable as shown in Figure 1a, with its three coordinate axes coincide with the corresponding axes of the turntable. The turntable precision was at the arc-second level and it can be controlled to rotate around different axes by computer instructions as shown in Figure 1b.

The phone was kept quiet with no motion firstly, and the gyroscope sampled for 1 h with the sampling frequency being 5 Hz. Then in order to verify the bias repeatability, the gyroscope sampled at different time during a day. It sampled every 2 h from 8 o’clock in the morning to 8 o’clock in the evening. The average value of the bias was calculated at each time by Equation (2). The bias distribution curve over time and its repeatability were estimated:

(2) $$\omega_{m}=\frac{1}{n}\overset{n}{\underset{i=1}{\sum }}\omega_{m}\left(i\right)$$

where *n* is the total sampling number at a time, and $\omega_{m}\left(i\right)$ is the *i*th gyroscope measurement.

The above steps validate the bias performances of a phone gyroscope, and then in order to obtain a precision rotating velocity, it is necessary to calibrate a gyroscope.

In this experiment, the turntable was controlled to rotate around its *X*, *Y*, and *Z* axes, respectively, for a few minutes, with a constant rotating velocity. The phone gyroscope sampled during the rotation and the average value was computed to represent the phone sensor measurement. Then, changing the rotating velocity and repeating above experiment till more than 20 samples were obtained and the installation error matrix $K_{\omega }$ can be estimated according to Equation (3) by using least squares method:

(3) $${\bar{\omega }}_{r}=K_{\omega }{\bar{\omega }}_{m}+b_{\omega }$$

where ${\bar{\omega }}_{r}$ is the rotating velocity read from the turntable, ${\bar{\omega }}_{m}$ means the average value of the gyroscope measurements during each rotation. $b_{\omega }$ represents the bias evaluated from the above steps.

#### 3.1.2. Accelerometer

According to [29], the measurement value of the acceleration sensor is modeled as:

(4) $$a_{r}=K_{a}a_{m}-b_{a}-w_{a}$$

where $a_{r}$ is the real acceleration of the sensor; $a_{m}$ is the measurement value; $K_{a}$ is the accelerometer installation error matrix; $b_{a}$ is the bias; and $w_{a}$ is the measurement error of the sensor.

According to Equation (4), it can be observed that if an accelerometer is available, its bias should be constant (can be modeled) and repeatable. In order to verify its stability, the mobile phone was put on a table with no motion. The accelerometer sampled for 1 h with the frequency being 5 Hz. Then, in order to verify the bias repeatability, the phone was sampled at different times in a day. It was sampled every 2 h from 8 o’clock in the morning to 8 o’clock in the evening, and then the average value of the bias was calculated by Equation (5):

(5) $$a_{m}=\frac{1}{n^{\prime }}\overset{n^{\prime }}{\underset{i=1}{\sum }}a_{m}\left(i\right)$$

where *n*′ is the total sampling number in an experiment, and $a_{m}\left(i\right)$ is the *i*th accelerometer measurement.

The above steps evaluate the bias performances of a phone accelerometer, and then in order to obtain a precision data, it is necessary to calibrate an accelerometer. In this experiment, the phone was installed on the tri-axes turntable as Figure 1 shown, and the turntable was controlled to rotate to a fixed angle around the phone’s three axes, respectively, and was kept still for a few minutes. The phone accelerometer sampled during the turntable was quiet and the average value was computed to represent the phone sensor measurement. Then, changing the rotation angle and repeating above experiment till more than 20 samples were obtained and the installation error matrix $K_{a}$ can be estimated according to Equation (6) by using least squares method:

(6) $$a_{r}=g\cdot cos\theta =K_{a}{\bar{a}}_{m}+b_{a}$$

where g is gravity’s influence on the accelerometer, and θ represents the sampling angle reading from the turntable. ${\bar{a}}_{m}$ means the average value of the accelerometer measurements during each test. $b_{a}$ represents the bias evaluated from the above steps.

**Figure 2:**
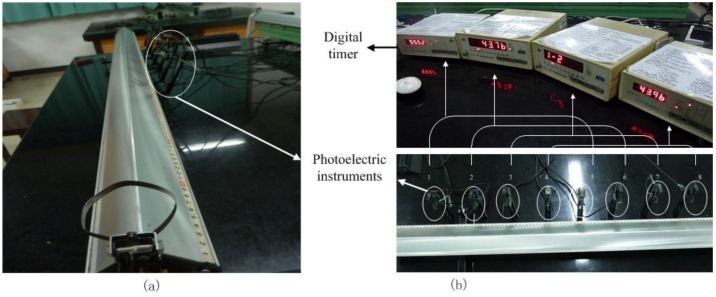
The experiment instruments for accelerometer accuracy estimation. (**a**) The QG-5 air track used in the experiment; (**b**) Four digital timer and eight photoelectric instruments.

Then, in order to test the accelerometer accuracy, the following estimation experiments were conducted. As Figure 2a shows, the experiment was designed based on a QG-5 air track. The air track is 2 m length with pinholes overall. A slide can move on the air track with little resisting force. The phone was fixed on a slide. The real accelerometer is obtained by two photoelectric instruments and a digital timer. In the experiment, eight photoelectric instruments and four digital timers were used as Figure 2b shown. Each digital timer connects with two photoelectric instruments. Once the slide moves across the photoelectric instruments, the digital timer can obtain the time and compute the accelerometer automatically. Then, the real acceleration is computed by Equation (7). The phone sampled during its movement, and the measurement accelerometer is the averaged value of the total sampling data:

(7) $$a_{r}=\frac{1}{4}\overset{4}{\underset{j=1}{\sum }}a_{r}\left(j\right)$$

where $a_{r}\left(j\right)$ is the accelerometer reading computed by the *j*th digital timer.

#### 3.1.3. Orientation Sensor

The orientation sensor of the mobile phone measures degrees of rotation that a device makes around its three physical axes. It is different from the physical sensors like the accelerometers and the gyroscopes. It belongs to a kind of software sensor. Its measurements integrate the gravity sensor and geomagnetic field sensor through a filtering algorithm. This kind of filtering fusion algorithm is quite mature and packaged into the mobile phone to measure the pitch, roll angle, and yaw angle.

The estimation method of orientation sensor accuracy was presented as follows. The turntable (Figure 1) was controlled to move around the *X*, *Y*, and *Z* axes, respectively, to different pitch, roll, and yaw. The turntable measured the real data of the attitude angle. The average measurement error value $\nu_{\rho }$ of the orientation sensor at different attitude angles could be calculated by Equation (8):

(8) $$\nu_{\rho }=\frac{1}{n_{\rho }}\overset{n_{\rho }}{\underset{i=1}{\sum }}(\rho_{m}\left(i\right)-\rho_{r})$$

where $n_{\rho }$ is the total sampling number of the orientation sensor when the sensor was static at a certain attitude angle. $\rho_{m}$ is the measurement value of the orientation sensor. $\rho_{r}$ is the reading of the attitude angle of the three-axis turntable.

#### 3.1.4. Pressure Sensor

Compared with the traditional gyroscope and accelerometer, the pressure sensor belongs to a new kind of sensor.

The experimenter placed tape on Point A at the top floor of a tall building, marking this point as a zero point, the absolute altitude being $h_{p0}$. Then the pressure sensor was mounted at the end of the tape. The tape was lowered slowly from Point A to the bottom of the building. In the process of descent, the pressure sensor recorded its absolute altitude value $h_{p}$ at a specific frequency. The true descent distance can be read by the tape, denoted as $h_{r}$. The height measurement error $\upsilon_{h}$ of the pressure sensor can be calculated by Equation (9):

(9) $$\upsilon_{h}=h_{r}-\left(h_{p}-h_{p0}\right)$$

### 3.2. Multi Phone Sensors Fusion-Based Visual Odometry (MFVO)

In this paper, the algorithm follows the classical framework used in the article [5] with its observation measurements are obtained from our previous method [13], and described briefly in this paper.

**Figure 3:**
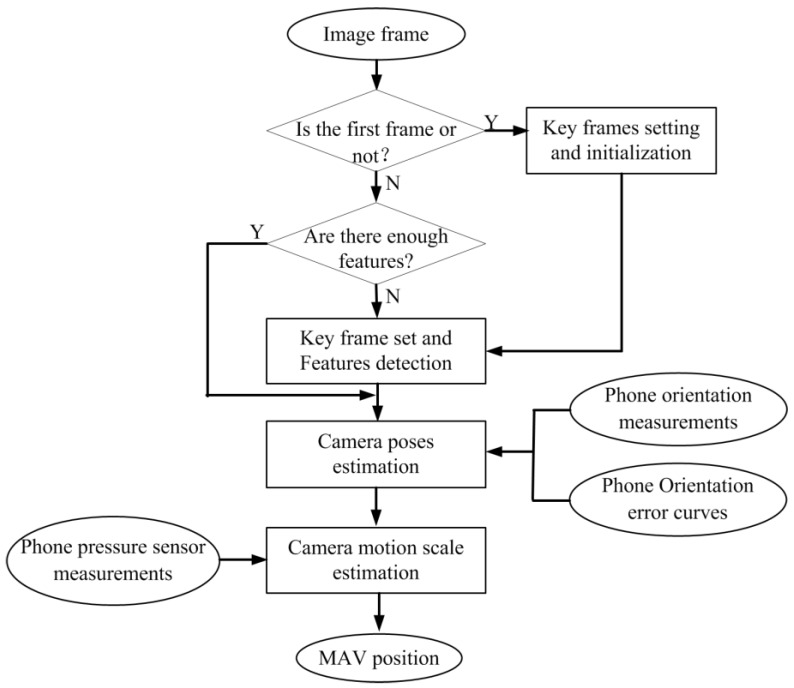
The framework of the algorithm.

The framework of the algorithm is shown in Figure 3. When the image is captured, if it is the first frame or the features for tracking is not enough, then the key frame is set and new features is detected. On the other hand, if the features are rich, the features are tracked and camera poses are estimated by integrating phone orientation measurements and error curves estimated in Section 3.1.3. Then, the camera motion scale is computed by fusing the visual information and pressure sensor measurements. The algorithm framework doses not reconstruct any environment map for quick computing. The localization accuracy is ensured by taking orientation and pressure sensors into consideration and is tested in the experiments and results. The key sections of the algorithm are detailed as follows.

Key frames setting and initialization: The first frame image is set as the key frame and establishes the database of the feature points to be tracked. With the camera movement, the database of the feature points to be tracked gradually became invalid, and the number of the feature points that can be matched is gradually reduced. When the number of matches is less than a threshold, the current frame is set to a new key frame, and the database of the features points is reestablished.

Camera poses estimation: The camera attitude information is estimated based on the eight-point algorithm [30] and fused with the orientation sensor based on the Kalman filter approach [31].

Camera motion scale estimation: The optimized camera attitude information is introduced into the estimation module of the translation, and the location of camera is solved combining with the pressure sensor.

## 4. Experiments and Results

### 4.1. Phone Sensors Accuracy Assessment

This paper takes Samsung Galaxy S3 as the specific research object. The built-in phone sensors performances were estimated and shown in the flowing results.

#### 4.1.1. Gyroscope

**Figure 4:**
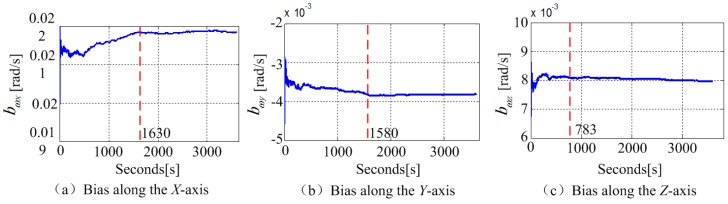
The change curve of the phone gyroscope bias over time.

**Figure 5:**
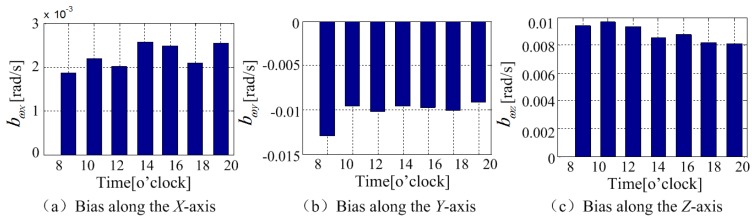
The change of the gyroscope bias within a day.

The bias distribution over time is shown in Figure 4. The bias of the MEMS gyroscope along *X* axis and *Y* axis had fluctuations at the first around 27 min and tended to stabilize with 0.0002 rad/s and 0.0001 rad/s deviation, respectively. The bias along *Z* axis tended to be stable until 13 min later with the deviation around 0.0001 rad/s. The average value changes in one day were shown in Figure 5. It can be observed from the total seven times experiments, the zero bias of the phone gyroscope were different at different times during a day. The standard deviations of the bias in *X*, *Y*, and *Z* axes were, respectively, 0.001 rad/s, 0.0003 rad/s, and 0.0005 rad/s. The results show that the gyroscope zero bias needs to be reevaluated and calibrated before every employment.

**Figure 6:**
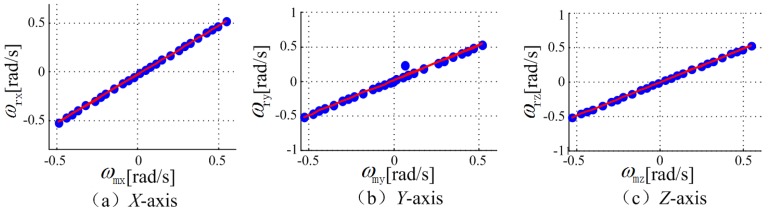
The gyroscope accuracy estimation results.

The gyroscope accuracy estimation results are shown in Figure 6. The horizontal axis is the phone gyroscope readings and the vertical axis represents the real rotating velocity obtained from the turntable. The points represent the corresponding samplings in different rotation experiments. The line is a fitting curve, from which it can be seen that the calibrated measurement of the phone gyroscope fits well with the real measurements. The mean error along the *X*-axis, *Y*-axis, and *Z*-axis are around 0.001 rad/s, 0.003 rad/s, and 0.002 rad/s respectively.

#### 4.1.2. Accelerometer

**Figure 7:**
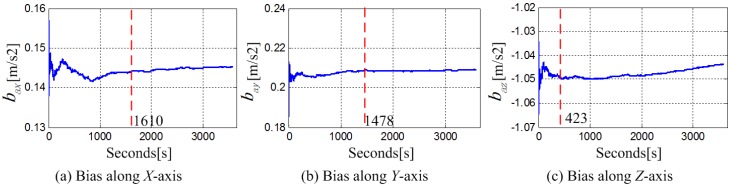
The change curve of the phone accelerometer bias over time.

**Figure 8:**
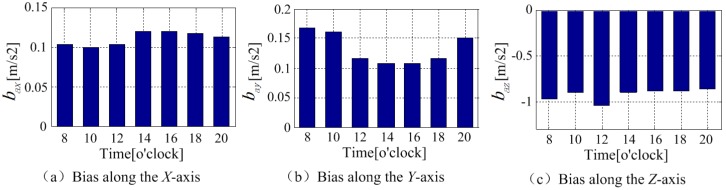
The change of the accelerometer zero bias within a day.

The bias stability of the built-in MEMS accelerometer was estimated as shown in Figure 7. As shown in the chart, the bias of the MEMS accelerometer along *X* axis and *Y* axis had fluctuations at the first 27 min and tended to stabilize with deviation around 0.0011 m/s^2^ and 0.0012 m/s^2^, respectively. The bias long *Z* axis was stable till around 7 min with the deviation around 0.0019 m/s^2^. Figure 8 shows the changes of the accelerometer bias in a day. It can be observed from the total seven experiments, the bias of the phone accelerometer were different at different time. The standard deviations of zero bias in the *X*, *Y*, and *Z* axes were, respectively, 0.0086 m/s^2^, 0.0264 m/s^2^, and 0.0628 m/s^2^, which represents that the accelerometer bias needs to be calibrated before every employment until it becomes stable.

**Figure 9:**
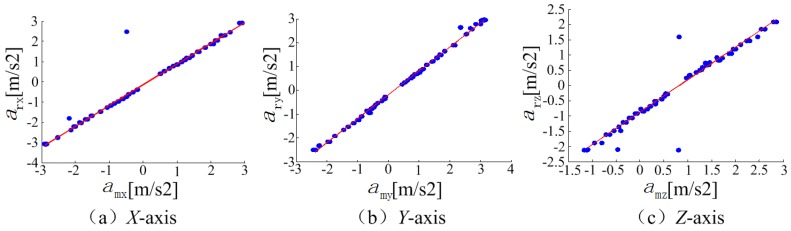
The accelerometer accuracy estimation results.

The accelerometer accuracy estimation results are shown in Figure 9. The horizontal axis is the phone accelerometer readings and the vertical axis represents the accelerometer obtained from the photoelectric instruments and digital timer. The points represent the corresponding samplings in different accelerometer. The red line is a fitting curve, from which it can be seen 98% points were lying on the line in the *X*-axis and *Y*-axis, and 92% points in the *Z*-axis. The mean error of the calibrated accelerometer measurements along the *X*-axis, *Y*-axis, and *Z*-axis are around 0.05 m/s^2^, 0.07 m/s^2^, and 0.15 m/s^2^, respectively.

#### 4.1.3. Orientation Sensor

**Figure 10:**
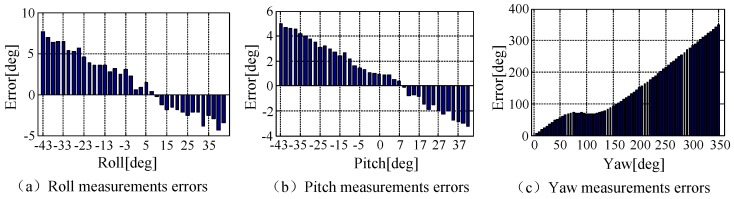
The error of the orientation sensor.

The error graph of the mobile phone orientation sensor was shown in Figure 10. From the chart it can be observed that the orientation sensor error of the mobile phone was not constant, but changed as the mobile phone attitude changed. For later convenient operation, the error table of the mobile phone orientation sensor was established in this paper according to experiment data. The effective range was the rolling angle/pitch angle [−45°, 45°], which could satisfy most of the actual smooth flight condition. A real-time lookup table can calibrate the measurement data of its orientation sensor. The yaw angle, which incorporated magnetic sensors, was easily vulnerable to the surroundings, especially in the complex magnetic experiment environment. Therefore, the measurement value of yaw angle was basically in a failure state in the process of locating.

#### 4.1.4. Pressure Sensor

**Figure 11:**
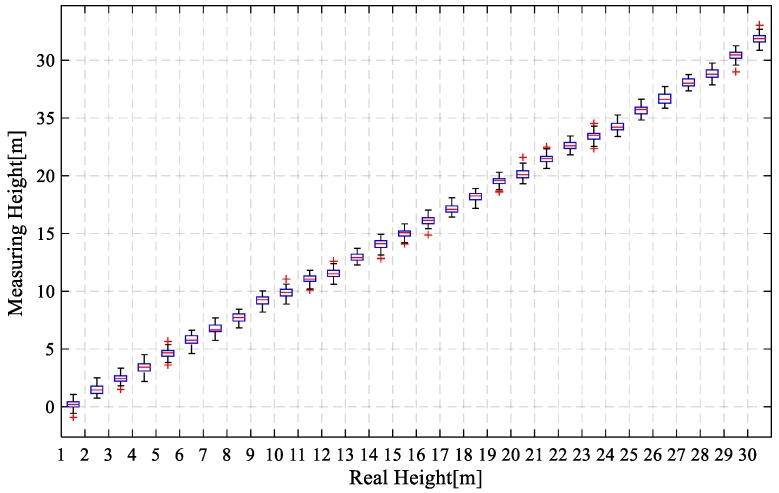
Height estimation results of the mobile phone pressure sensor.

The pressure sensor error curve was shown in Figure 11. In the figure, the abscissa axis represented the real descent height of the mobile phone, and the vertical axis represented the measurement height of the mobile phone pressure sensor. By integrating sampling data, the average value of measurement error of the mobile phone pressure sensor was 0.28 m, and the standard deviation was 0.026 m. It can be judged by experiments that the relative height measurement error of pressure sensors was continuously showing a decreasing trend, and the measurement error provided by the pressure sensor was about 30 cm, which had a high reliability.

### 4.2. Algorithm Accuracy Assessment

#### 4.2.1. Parrot AR Drone Experiment

In this part, a comparison indoor experiment was conducted. Since the open datasets (such as TUM, KITTI) provide no orientation and pressure sensor measurements which are necessary in our proposed algorithm. The proposed algorithm cannot run based on the public datasets. After searching for many methods, we choose TUM_PTAM [28] algorithm which is not the latest but one of the most classical solutions. This algorithm is open source and can be realized without the homemade specific hardware. TUM_PTAM algorithm enables a low-cost quadcopter to navigate autonomously in GPS-denied environments. The TUM_PTAM estimates small UAV poses by integrating the PTAM algorithm [12] and IMU sensor measurements based on an extended Kalman filter (EKF).

**Figure 12:**
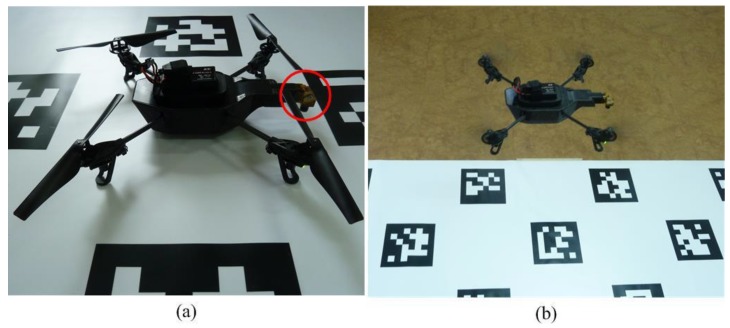
The platform for the comparison experiment. (a) The AR Drone with its camera looking downward; (b) The flying platform.

As a platform, we chose a Parrot AR drone, the same hardware employed in the TUM_PTAM system while a revision was made: in our approach, the camera needs to aim downward. Therefore, the first camera of the AR drone was reinstalled to face downward (Figure 12a) and the relative transformation matrix used in TUM_PTAM approach was revised. The revision was tested to have no influence to the TUM_PTAM algorithm accuracy performance.

**Figure 13:**
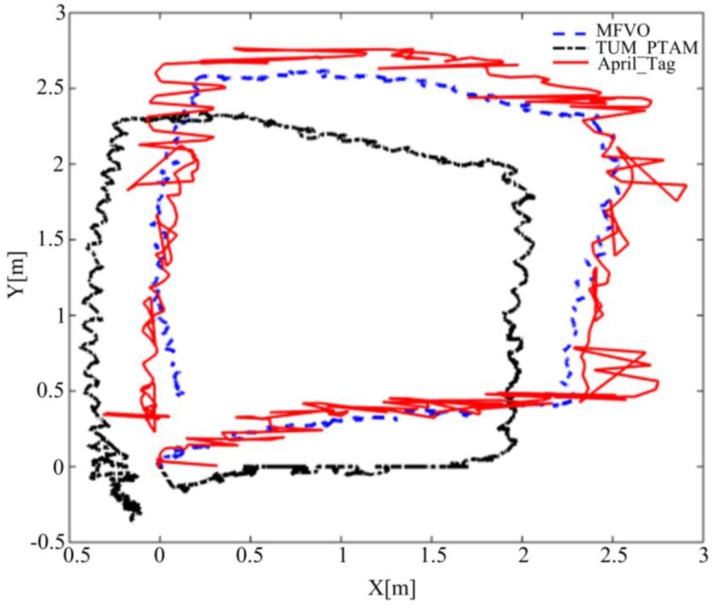
Paths comparison of the TUM_PATM and the proposed algorithm (MFVO).

The comparison experiment was conducted in an indoor environment, because the outdoor environment is under the influence of light and wind, which may lead additional localization errors. The AR drone was controlled to move a square trajectory at a fixed height of 1m. The ground truth was computed by the AprilTag algorithm [32] (Figure 12b). In order to ensure enough features for vision algorithms, extra artificial textures were provided. Figure 13 illustrates three different paths computed by different algorithms, where the red solid, blue dashed and black dash-dotted lines represent paths computed by AprilTag, the proposed algorithm and the TUM_PTAM [28], respectively.

The localization root-mean-square error (RMSE) of the proposed algorithm is 0.42 m, while the TUM_PTAM is around 0.71 m. The tracking errors TUM_PTAM are mainly produced by the 3D outliers in the environment map. The proposed algorithm efficiently copes with this problem by estimating the camera poses relative to the key frames rather than the environment map, while the location accuracy is improved by fusing multi-sensor. However, from the figure it can be seen that the April_Tag path is jumping while the drone is in stable flight. According to [32], this is because when the drone flies with its camera looking downward, more than one tag can be detected in the image, and the April_Tag algorithm choses one of the tags to estimate the drone position randomly. With the drone moving, the tag for localization switches to another one, which results in the localization value jumps from one point to another. However, from the figure it can be found that the path estimated by our algorithm is much closer to April_Tag path than that of TUM_PTAM.

#### 4.2.2. Flying Experiment in the Wheat Fields

**Figure 14:**
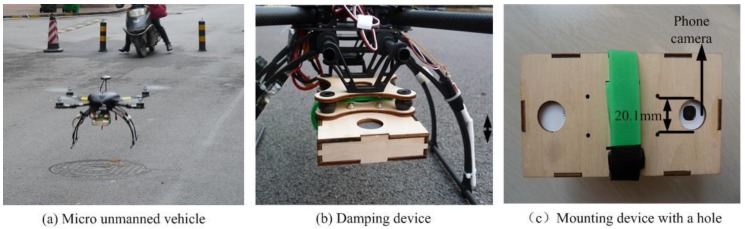
Experiment equipment of the outdoor experiments.

An XAircraft 650 quadrotor (Figure 14a) was selected as the outdoor experiment platform. The control of aircraft was developed based on the open source flight control. In the outdoor experiment, Samsung Galaxy S3 was installed in the front of the aircraft, with the camera down. As shown in Figure 14b, the damping device was specially designed for the mobile phone. The device can obviously relieve the impact of the flight vibration on the sensor. In order to facilitate the work of collecting images by the mobile phone, a hole with a diameter of 20.1 mm was opened for the camera to ensure image acquisition during the flight (Figure 14c). In the experiment, the pilot manually controlled the UAV to fly and the airborne GPS signal recorded the flight trajectory while the mobile phone sensor was sampling the current flight status data and images. In the outdoor experiment, the algorithm was run in the offline case. The flight data was processed based on the NUC board to verify the location accuracy of the algorithm by comparing with GPS data. For data collection, the mobile phone sensor recording app was developed. The video stream was stored in .mp4 file format. The sensor data was stored in .db format data file, which was stored in the mobile’s SD card. The phone sensors sampled data at the frequency set in Table 1 and the algorithm parameters were set in Table 2.

**Figure 15:**
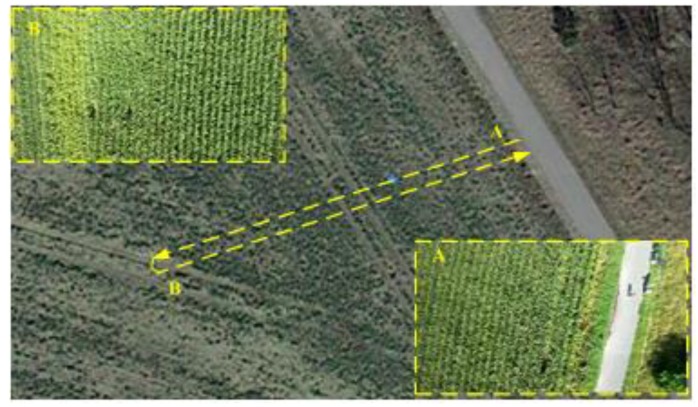
Wheat field experiment environment.

The first outdoor experiment was conducted in the wheat field environment shown in Figure 15. Since the wheat fields have rich texture information, it could ensure the accuracy of visual information. In the experiment, the rotor UAV mainly moved along a straight line slowly from Point A to Point B. The onboard images were shown in Figure 15. The pilot controlled the UAV at 35 m high by the remote-controller.

**Figure 16:**
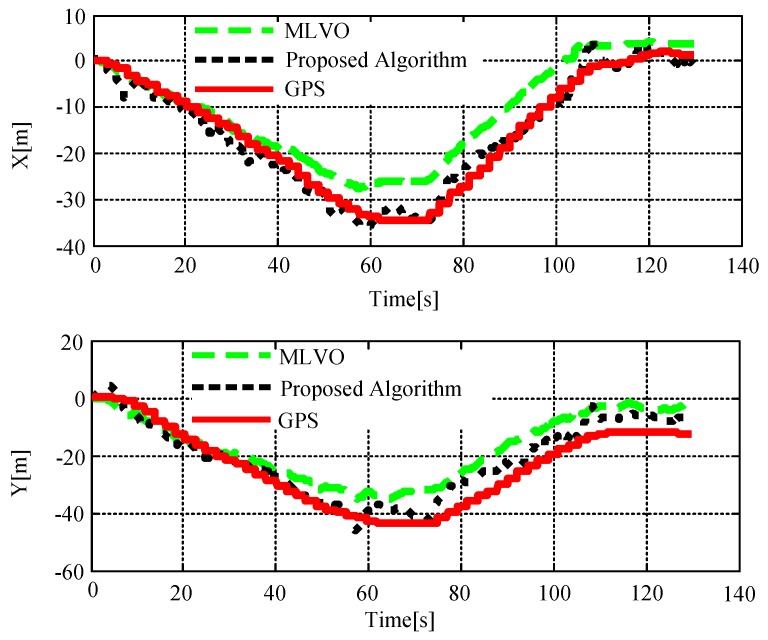
Contrast diagram of flight test trajectories.

Figure 16 shows the flight test trajectory in the wheat field. The red solid line indicated the real flight trajectory provided by the airborne GPS. The black dot line was the flight trajectory calculated by the algorithm in this paper. The green dashed line was our previous method MLVO [33]. It can be seen from the picture. In the early stages of flight, the track curve of MLVO and the proposed algorithms were basically in the phase of overlapping. With the aircraft moving on, the curve of these two algorithms began to deviate because of the platform shaking, matches mistakes, and so on. However, the RMSE of MLVO algorithm was 5.34 m in the *X* direction and 8.22 m in the *Y* direction, while the proposed algorithm in this paper was 2.28 m in the X direction and 4.54 m in the *Y* direction. From the results, it can be observed that comparing MLVO method, the proposed algorithm improves the localization accuracy by 30.41%. From the figure it can be observed that the proposed algorithm localization error increases with the distances of the flight, which is decided by the map-less VO framework, around 8% of the distances in the outdoor environments.

#### 4.2.3. Flying Experiment in the Urban Environment

The second flying experiment was carried out in an urban environment shown. Here, we aimed to test the applicability of the proposed method in a city environment under a variety of challenging factors, such as moving objects, large height changes. In this experiment, the MAV was manually instructed to take off and fly a trajectory of about 150 m at an altitude of 25 m.

**Figure 17:**
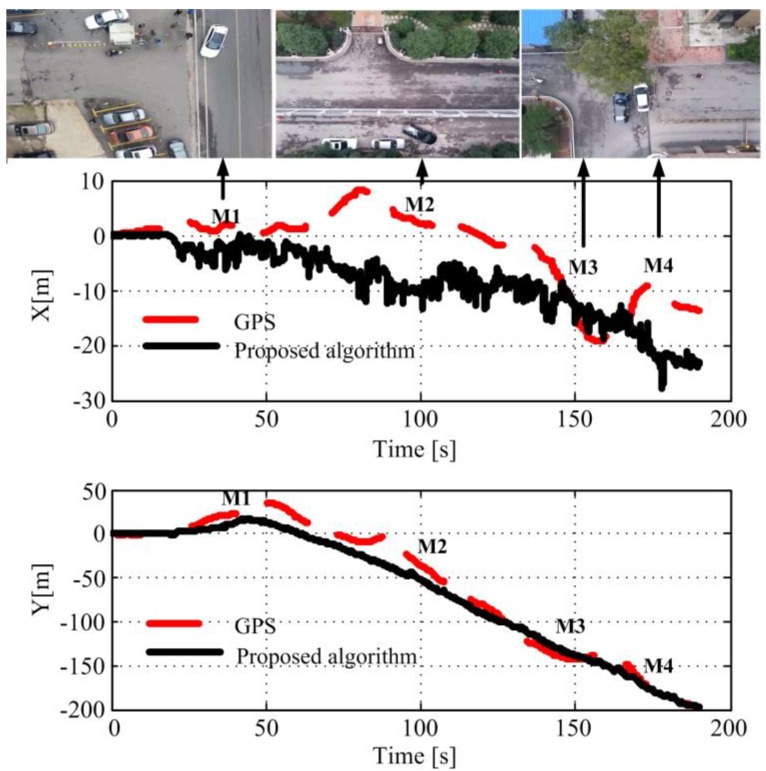
Localization results in the urban environment.

Figure 17 illustrates the flying paths recovered by the proposed methods and GPS records, where the black solid line represents the MFVO path, and the red dashed line is the GPS trajectory. From the results it can be observed that the proposed localization algorithm can fit the GPS trajectory at the first 25 m. However, while the flight is moving on, the algorithm began to drift around 15.8 m in the *X*-direction and 8.1 m in the *Y*-direction due to the influence of wind and the ground moving vehicles.

From the paths it can be observed that the localization accuracy was not satisfying in the urban environment. The localization accuracy drifts away at Point M1, M2, M3, and M4. Observing the onboard frames at that time it can be found that there were moving cars in the street as shown in Figure 17. The moving objects in the view influence the visual tracking efficiency, which results in the tracking failure. On the other hand, the path recovered along the *Y* direction is much better than that along the *X* direction. After flying 200 m, the localization RMSE is around 8.1 m, which is around 4.05% of the flight distance.

## 5. Discussion and Conclusions

This paper tries to explore a real-time UAV autonomous location method based on the phone sensors by taking advantages of the mobile device which features high integration, lightness, and convenience. This coincides with the demand of the small UAV platform for loads, which enabled the small UAV to work in areas with unstable GPS signals, such as indoors and the tunnel. By taking the Samsung Galaxy S3 as the specific research object, the measurement accuracy of the built-in MEMS accelerometers, gyroscopes, pressure sensors, and orientation sensors were evaluated. The error results showed that the bias stability and repeatability of MEMS accelerometer and gyroscope were poor. The biases were unstable at the first around 27 min when it was operating, which means that it is quite necessary to carry out a pre-calibration and filtering work before every employment. After calibration, the phone pressure sensor and the orientation sensor have good precision and can be used directly to measure the height and attitude angle of the platform steadily.

On the other hand, the proposed algorithm is high efficiency, operates in real-time, and occupies less memory space in the indoor localization experiment compared with the TUM_PTAM algorithm. The localization path of the proposed algorithm is much fits with the reference April_Tag path. In the outdoor experiment, the proposed algorithm can track the GPS path with its localization RMSE around 4%–8% of the travelling distances. However, through analysis of the outdoor experiment results, the proposed algorithm was mainly inclined to be influenced by the ground moving objects. How to improve the accuracy under dynamic environments remains a problem and need to be studied in the future work.

The most concerning performance in the article is the localization accuracy. Therefore, different accuracy estimation experiments were designed and conducted in the manuscript (Section 4.1 shows phone sensor accuracy assessments and Section 4.2 shows algorithm accuracy assessments). The other important performance is the phone computation time, which has been investigated in many papers [22,23]. In our future work, the computing time and the consuming-resources of the algorithm will be considered. Additionally, from recent research works [22,34,35] it can be observed that how controlling a UAV-based on phone sensors remains a problem. The control strategy based on the proposed algorithm will be investigated in future work.

## Figures and Tables

**Table 1:** The sampling frequency of the mobile phone sensors.

Sensors	Sampling Frequency [Hz]
Camera	30
Orientation sensor	20
Pressure sensor	40
Accelerometer	50
Gyroscope	50

**Table 2:** The parameter settings of the algorithm.

The Name of Parameters	Parameter Setting
Camera resolution [pixels]	640×480
Image segmentation *N*	4
Orientation threshold $T_{c}$	0.05
Manhattan distance $T_{d}$	0.2
Number of maximum feature	200
Minimum features for keyframe selection	100
